# Diagnostic Usefulness of Spiroergometry and Risk Factors of Long COVID in Patients with Normal Left Ventricular Ejection Fraction

**DOI:** 10.3390/jcm12124160

**Published:** 2023-06-20

**Authors:** Katarzyna Gryglewska-Wawrzak, Agata Sakowicz, Maciej Banach, Ibadete Bytyçi, Agata Bielecka-Dabrowa

**Affiliations:** 1 Heart Failure Unit, Department of Cardiology and Congenital Diseases of Adults, Polish Mother’s Memorial Hospital Research Institute (PMMHRI), 93-338 Lodz, Poland; maciejbanach77@gmail.com (M.B.); agatbiel7@poczta.onet.pl (A.B.-D.); 2 Department of Medical Biotechnology, Medical University of Lodz, 90-752 Lodz, Poland; agata.sakowicz@gmail.com; 3 Department of Preventive Cardiology and Lipidology, Medical University of Lodz, 93-338 Lodz, Poland; 4 Department of Public Health and Clinical Medicine, Umeå University, 901 87 Umeå, Sweden; i.bytyci@hotmail.com; 5 Clinic of Cardiology, University Clinical Centre of Kosova, 10000 Prishtina, Kosovo

**Keywords:** COVID-19, long COVID syndrome, exercise intolerance, body mass compartments

## Abstract

The emergence of the Coronavirus Disease 2019 (COVID-19) pandemic has brought forth various clinical manifestations and long-term complications, including a condition known as long COVID. Long COVID refers to a persistent set of symptoms that continue beyond the acute phase of the disease. This study investigated the risk factors and the utility of spiroergometry parameters for diagnosing patients with long COVID symptoms. The 146 patients with severe acute respiratory syndrome coronavirus 2 (SARS-CoV-2) infection with normal left ventricular ejection fraction and without respiratory diseases were included and divided into two groups: the group demonstrating long COVID symptoms [n = 44] and the group without long COVID symptoms [n = 102]. The clinical examinations, laboratory test results, echocardiography, non-invasive body mass analysis, and spiroergometry were evaluated. ClinicalTrials.gov Identifier: NCT04828629. Patients with long COVID symptoms had significantly higher age [58 (vs.) 44 years; *p* < 0.0001], metabolic age [53 vs. 45 years; *p* = 0.02)], left atrial diameter (LA) [37 vs. 35 mm; *p* = 0.04], left ventricular mass index (LVMI) [83 vs. 74 g/m^2^, *p* = 0.04], left diastolic filling velocity (A) [69 vs. 64 cm/s, *p* = 0.01], the ratio of peak velocity of early diastolic transmitral flow to peak velocity of early diastolic mitral annular motion (E/E’) [7.35 vs. 6.05; *p* = 0.01], and a lower ratio of early to late diastolic transmitral flow velocity (E/A) [1.05 vs. 1.31; *p* = 0.01] compared to the control group. In cardiopulmonary exercise testing (CPET), long COVID patients presented lower forced vital capacity (FVC) [3.6 vs. 4.3 L; *p* < 0.0001], maximal oxygen consumption measured during incremental exercise indexed per kilogram (VO_2max_) [21 vs. 23 mL/min/kg; *p* = 0.04], respiratory exchange ratio (RER) [1.0 vs. 1.1; *p* = 0.04], forced expiratory volume in one second (FEV1) [2.90 vs. 3.25 L; *p* = 0.04], and a higher ratio of forced expiratory volume in one second to forced vital capacity (FEV1/FVC%) [106 vs. 100%; *p* = 0.0002]. The laboratory results pointed out that patients with long COVID symptoms also had a lower rate of red blood cells (RBC) [4.4 vs. 4.6 × 10^6^/uL; *p* = 0.01]; a higher level of glucose [92 vs. 90 mg/dL; *p* = 0.03]; a lower glomerular filtration rate (GFR) estimate by Modification of Diet in Renal Disease (MDRD) [88 vs. 95; *p* = 0.03]; and a higher level of hypersensitive cardiac Troponin T (hs-cTnT) [6.1 vs. 3.9 pg/mL; *p* = 0.04]. On the multivariate model, only FEV1/FVC% (OR 6.27, 95% CI: 2.64–14.86; *p* < 0.001) independently predicted the long COVID symptoms. Using the ROC analysis, the FEV1/FVC% ≥ 103 was the most powerful predictor of spiroergometry parameters (0.67 sensitive, 0.71 specific, AUC of 0.73; *p* < 0.001) in predicting the symptoms of long COVID. Spiroergometry parameters are useful in diagnosing long COVID and differentiating it from cardiovascular disease.

## 1. Introduction

Severe acute respiratory syndrome Coronavirus 2 (SARS-CoV-2) is a highly transmissible and pathogenic coronavirus that emerged in late 2019 and has caused a pandemic of acute respiratory disease, named Coronavirus Disease 2019 (COVID-19), which threatens human health and public safety. At the end of 2019, a novel coronavirus designated SARS-CoV-2 emerged in the city of Wuhan, China, and caused an outbreak of unusual viral pneumonia [1]. The World Health Organization declared COVID-19 a pandemic in March 2020 [2]. Symptoms include coughing, fever, and shortness of breath. Common symptoms are fever, cough, sore throat, dyspnea, smell and taste disturbances, weakness, malaise, and muscle pain [3]. The SARS-CoV-2 infection is not limited to the respiratory system. It may trigger an excessive immune response known as a cytokine storm, which can lead to multiple organ failure and death [4]. Cardiovascular complications can be a significant contributor to the mortality associated with this disease. The mechanisms of cardiovascular injury caused by SARS-CoV-2 infection have not been fully elucidated, but it is speculated that SARS-CoV-2 affects the cardiovascular system through multiple mechanisms, including direct injury, downregulation of angiotensin-converting enzyme 2 (ACE2), immune injury, hypoxia injury, and psychological injury [5]. Patients with COVID-19 can present with dyspnea, chest pain, arrhythmias, and acute myocardial injury [6]. Studies have estimated that 4.5% to 36.6% of all COVID-19 patients continue to suffer from symptoms more than 3 months post-infection [7]. This condition is defined as long COVID syndrome [8]. The analysis of 153.760 individuals in national healthcare databases from the US Department of Veterans Affairs, with comparison to over 10 million contemporary and historical controls, reported an important expansion in the incidence of cardiovascular disease in surviving patients and a 55% increase in combined cardiovascular outcome 1 year after COVID-19. Additionally, increased risk was observed even in non-hospitalized patients, with risk related to the severity of the acute infection [9]. Dyspnea, fatigue, chest pain, muscle pain, cognitive impairment, taste and smell disturbances, and exercise intolerance are the most frequent symptoms of long COVID. Exercise capacity is defined as the maximum ability of the cardiovascular system to deliver oxygen to exercising skeletal muscle. It is determined by pulmonary gas exchange, cardiovascular performance, and skeletal muscle metabolism [10]. Cardiopulmonary exercise testing (CPET) provides an evaluation of exercise capacity and assessment of integrative exercise responses involving the pulmonary, cardiovascular, hematopoietic, neuropsychological, and skeletal muscle systems [11]. Therefore, we sought to determine the utility of CPET parameters in differentially diagnosing patients with long COVID syndrome.

The aim of this study was to investigate the risk factors and assess the utility of spiroergometry parameters in differentially diagnosing patients presenting symptoms (dyspnea, fatigue, pain in the chest, muscle pain, cognitive impairment, taste, and smell disturbances) persisting for a few months after recovery from SARS-CoV-2 infection (symptoms of long COVID).

## 2. Materials and Methods

### 2.1. Basic Characteristics

From the Department of Cardiology, 146 consecutive patients recovering from SARS-CoV-2 infection three to six months after a confirmed diagnosis were recruited for this study. Patient inclusion in the analysis was performed based on the existence of the exclusion criteria at the study start (three to six months after infection). A random sample from the electronic medical record was reviewed independently and in duplicate to validate the research strategy. The subjects were hospitalized in the Department of Cardiology and Congenital Heart Diseases of Adults between December 2020 and December 2021. The subjects were divided into the two following groups: the group demonstrating long COVID symptoms (i.e., suffering from one of the following: dyspnea, fatigue, pain in the chest, muscle pain, cognitive impairment, taste or smell disturbances) [n = 44] and the group without long COVID symptoms [n = 102]. There were no differences between groups in pharmacological treatment. All subjects gave written informed consent to participate in this study. Patients performed CPET on the ergometer. The exclusion criteria were as following: unstable arterial hypertension; unstable angina; acute pulmonary embolism; diagnosis of heart failure or typical symptomatic heart failure; left ventricular ejection fraction (LVEF) < 50%; past myocardial infarction; unstable heart rhythm disorders; acute myocarditis or pericarditis; active endocarditis; advanced atrioventricular block; diagnosed cardiomyopathy (hypertrophic, dilated, restrictive, postpartum, tachyarrhythmic); stroke, transient ischemic attack, history of intracerebral bleeding; severe hyper- and hypothyroidism; pregnancy or lactation; chronic kidney disease (stage IV and V according to the National Kidney Foundation) and dialysis treatment; documented neoplastic process; the patient’s inability to cooperate and/or give informed consent to participate in a research; alcohol and drug abuse; active autoimmune disease; taking immunosuppressants, cytostatic drugs, glucocorticosteroids, or antiretroviral drugs; a history of bone marrow or other organ transplant; treatment with blood products within the last 6 months; active systemic infection; hepatitis B virus (HBV), hepatitis C virus (HCV), or human immunodeficiency virus (HIV) carrier or positive for hepatitis B surface antigen (HBsAg) or antibodies to HCV; surgery or a serious injury in the last month; physical disability that prevents the performance of a spiroergometric test; patients who did not express their informed consent to participate in this study. 

This study is in compliance with the Declaration of Helsinki and was approved by the Polish Mother’s Memorial Hospital Research Institute (PMMHRI-BCO.75/2020).

### 2.2. Laboratory Tests

Diagnostic blood samples were collected from each patient. The samples were obtained by needle puncture and withdrawn by suction through the needle into a vacuum blood collection system. Laboratory tests were performed in the hospital laboratory following a minimum 12-h period after the last meal. Routine laboratory tests included liver function [the alanine aminotransferase (ALT) and aspartate transaminase (ASP)] parameters and renal function [creatinine, glomerular filtration rate (GFR) estimate by Modification of Diet in Renal Disease (MDRD)] parameters, urea level, serum natrium (Na) and potassium (K) level, C-reactive protein (CRP), glucose level, lipoprotein profile: total cholesterol (TC), low-density lipoprotein (LDL), high-density lipoprotein (HDL) and triglycerides (TG), haematology, and D-dimer. In addition, the analysis of N-terminal pro-B-type natriuretic peptide (NT-proBNP) and high-sensitivity cardiac troponin T (hs-cTnT) was conducted. 

### 2.3. Echocardiography

The patients underwent transthoracic echocardiography (TTE) using the Vivid E95 system (GE Healthcare, Chicago, IL, USA). Quantitative measures were performed in accordance with current guidelines [12]. We calculated left ventricular dimensions in the end diastole: left ventricular internal diameter (LVID d), interventricular septum (IVS d), and left ventricular posterior wall (LVPW d). Left ventricular volume (LV) and ejection fraction (EF) were measured by the quantitative 2-dimensional biplane modified Simpson method from a 4- and 2-chamber view. The 2-dimensional maximal left atrial volume (LAV) was determined based on the apical 2- and 4-chamber views at end-systole without foreshortening, using a biplane modified Simpson’s method excluding the LA appendage and pulmonary vein confluences [13]. Each LAV was indexed by body surface area (LAVi). The LV mass index (LVMI) was calculated by dividing the LV mass (in grams) by a body size variable, such as body surface area. Residual echocardiographic parameters analyzed were: maximal early (E) and late (A) transmitral velocities; ratio of early transmitral peak velocity to early diastolic peak annular velocity (E/E′); ratio of early to late diastolic transmitral flow velocity (E/A); and deceleration time (Dec) and acceleration time (Ats). Global peak systolic strain (GLPS) was obtained using speckle-tracking echocardiography [14]. We also assessed the ascending aorta (AA), aortic bulb (AB), main pulmonary artery (MPA), and inferior vena cava (IVC) diameters. The right ventricular (RV) functional measure was tricuspid annular plane systolic excursion (TAPSE) and tissue Doppler echocardiography (TDE) [15]. Additionally, we obtained the right atrial volume (RA) and distal right ventricular outflow tract (RVOT d).

### 2.4. Spiroergometry

Symptom-limited cardiopulmonary exercise testing (CPET) was performed on an electromagnetically braked upright cycle ergometer, Bike M (CORTEX Biophysik GmbH, Leipzig, Germany), with a metabolic gas analyzer, METALYZER 3B (CORTEX Biophysik GmbH, Leipzig, Germany), using the MetaSoft Studio application software of CORTEX systems. Prior to exercise, basic spirometry was performed. We recorded forced vital capacity (FVC) and forced expiratory volume in one second (FEV1). Additionally, the FEV1/FVC ratio (Tiffeneau index) was obtained. Additionally, we evaluated forced expiratory flow over the middle half of the FVC (FEF 25–75) [16,17]. CPET on a bicycle ergometer was conducted with an additional continuous 12-lead electrocardiogram (ECG), heart rate (HR), oxygen saturation (SpO_2_), and non-invasive blood pressure (NIBP) monitoring. The following parameters are important in the interpretation of CPET. Oxygen uptake (VO_2_) is calculated from the difference between the volume of O_2_ in the inhaled and exhaled air during exercise per unit of time, and in a steady state, it is equal to metabolic O_2_ consumption. Peak VO_2max_ represents the highest attainable VO_2_ for a subject [18]. We also assessed other valuable CPET parameters. These derived measurements include respiratory exchange ratio (RER), oxygen uptake at anaerobic threshold (VO_2_AT), and the minute ventilation/carbon dioxide production slope (VE/VCO_2_ slope) [19].

### 2.5. Body Mass Analysis

The Segmental Body Composition Analyzer (Tanita Pro, Tokyo, Japan) is a device for non-invasive body mass analysis. This equipment provides estimated values for each measured value by the Dual-energy X-ray absorptiometry (DXA) method, an estimated value for the total body water measured value by the dilution method, and an estimated value for the visceral fat rating by the Magnetic Resonance Imaging (MRI) method using the Bioelectrical Impedance Analysis (BIA) method [20]. After gender, age, and height information had been entered into the device, participants were asked to stand barefoot in a stable position. The analyzer provides separate mass readings for different segments of the body and estimates total and regional fat mass (FM) and fat-free mass (FFM). Additionally, total body water (TBW), intracellular water (ICW), and extracellular water (ECW) were measured. Additionally, we examined the association between ECW/TBW, defined as the ECW/TBW% ratio, and basal metabolic rate (BMR) [21].

### 2.6. Statistical Analysis

The STATISTICA 13.1 software package (StatSoft, Cracow, Poland) was used for analysis. The concordance of the normal distribution of all variables was calculated with the Shapiro–Wilk test. To compare the 2 groups, the Student’s *t*-test for continuous variables with a normal distribution and the Mann–Whitney U test for non-normally distributed variables were used. Predictors of the long COVID symptoms were identified using univariate analysis and the multivariate logistic regression method. The receiver operating characteristic (ROC) analyses were performed, and the sensitivity and specificity were determined. To reduce the bias with age between the long COVID group and the non-long COVID population, one-to-one nearest-neighbor propensity score matching was used. The parameters that met the following criteria: (1) they were statistically significant in univariate analysis and (2) the area under the ROC curve (AUC) was at least 0.630 were qualified for the multivariate model. Only the 6 parameters met both criteria, i.e., age, metabolic age, A, E/A, E/E’, and FEV1/FVC%. For these parameters, the cut-off points based on the Youdan index of the ROC curve were determined, i.e., age < 54 [years], metabolic age < 49 [years], A < 68 [cm/s], E/A < 1.3, E/E’ < 7.15, and FEV1/FVC% > 103. As the correlations between A [cm/s] and E/A as well as between A [cm/s] and E/E’ were observed (R spearman = −0.54; *p* < 0.0001 and R spearman = 0.34; *p* = 0.002, respectively), in the multivariable analysis only age < 54 [years], metabolic age < 49 [years], A < 68 [cm/s], and FEV1/FVC% > 103 were included. The results for the chosen parameters were transformed into dichotomous variables based on the Youden point. A chi-square test was used to compare dichotomous variables between the groups. In analyses, a *p*-value < 0.05 was considered statistically significant.

## 3. Results

### 3.1. Evaluation of Basic Characteristics

In this study, 146 consecutive patients were enrolled. The subjects were divided into two groups: the group demonstrating long COVID symptoms [n = 44] and the group without long COVID symptoms [n = 102]. Subjects in this study group had significantly higher ages [median 58.0 (IQR: 48.0–67.0) vs. 44.5 (IQR: 31.0–53.0), *p* < 0.0001]. Statistically significant differences were not observed regarding body mass index (BMI), body surface area (BSA), systolic and diastolic blood pressure (SBP and DBP), and heart rate (HR). The data are presented in Table 1.

### 3.2. Evaluation of Laboratory Tests

Patients with symptoms presented with higher levels of hs-cTnT [median 6.10 (IQR: 3.20–9.00) vs. 3.90 (IQR: 3.00–6.20) pg/mL, *p* = 0.04] and glucose [median 91.50 (IQR: 86.00–99.00) vs. 90.00 (84.50–93.00) mg/dL, *p* = 0.03] in comparison to controls. Red blood cell (RBC) concentration and GFR were lower in the study group compared to patients without symptoms [median 4.42 (IQR: 4.13–4.80) vs. 4.62 (IQR: 4.32–5.08) 10^6^/uL, *p* = 0.01; median 88.25 (IQR: 76.10–98.80) vs. 94.90 (IQR: 81.80–111.30) mL/min/1.73 m^2^, *p* = 0.03, respectively]. Statistically significant differences were not observed regarding other biochemical parameters. We showed the results in Table 2.

### 3.3. Evaluation of Echocardiography

Subjects with symptoms presented with higher LA, LVMI, A velocity, and E/E’ [median 37.00 (IQR: 34.00–42.00) vs. 35.00 (IQR: 32.00–39.00) mm, *p* = 0.04; median 83.00 (IQR: 71.00–98.00) vs. 74.00 (IQR: 61.00–98.00) g/m^2^, *p* = 0.04; median 69.00 (IQR: 60.00–83.00) vs. 64.00 (IQR: 51.00–74.00) cm/s, *p* = 0.01; median 7.35 (IQR: 6.00–8.95) vs. 6.05 (IQR: 5.50–7.10), *p* = 0.01, respectively] compared to control group. E/A was decreased in the study group [median 1.05 (IQR: 0.80–1.22) vs. 1.31 (0.95–1.67), *p* = 0.01] in comparison to patients without symptoms. We did not observe statistically significant differences regarding other echocardiographic parameters. Table 3 contains the attached data.

### 3.4. Evaluation of Spiroergometry

**Figure 1:**
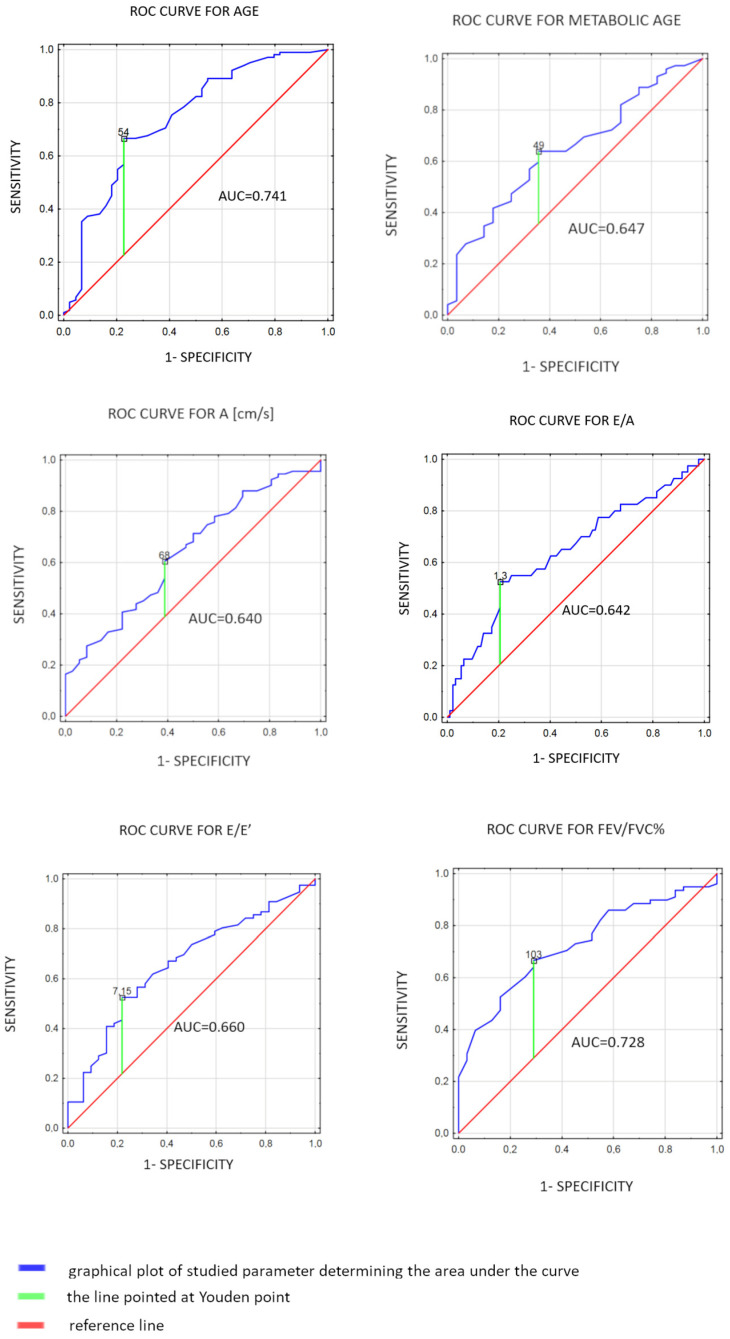
Receiver operating characteristic curves (ROC) for spiroergometric parameters in patients with long COVID symptoms. A—late diastolic filling velocity; E/A—ratio of early to late diastolic transmitral flow velocity; E/E’—ratio of peak velocity of early diastolic transmitral flow to peak velocity of early diastolic mitral annular motion as determined by pulsed wave Doppler; FEV1/FVC—ratio of forced expiratory volume in one second to forced vital capacity.

HR max and FEV1/FVC (%) were statistically greater in the study group [146.63 (±20.46) vs. 132.87 (±33.61), *p* = 0.006; median 106.50 (IQR: 99.00–112.00) vs. 100.00 (IQR: 90.00–105.00)%, *p* = 0.0002, respectively] compared to controls. Patients with symptoms also had lower FEV1, FVC (L), RER, and VO_2max_ than subjects without symptoms [median 2.90 (IQR: 2.55–3.54) vs. 3.25 (IQR: 2.79–3.71) L, *p* = 0.04; 3.65 (±0.89) vs. 4.32 (±1.02) L, *p* < 0.0001; median 1.08 (IQR: 1.01–1.11) vs. 1.10 (IQR: 1.05–1.12), *p* = 0.04; median 21.00 (IQR: 16.00–25.00) vs. 23.00 (IQR: 19.00–29.00) mL/min/kg, *p* = 0.04, respectively]. There were no significant differences between exercise time, level of effort, SBP and DBP max, FVC (%), FEV1/FVC, FEF 25–75, VO_2_AT, peak VO_2max_, and VE/VCO_2_ slope [*p* > 0.05 for all]. The spiroergometry parameters predicted long COVID-19 symptoms, with FEV1/FVC% ≥ 103 as the strongest predictor (0.67 sensitive, 0.71 specific, with an AUC of 0.73; *p* < 0.001, Figure 1). Results are shown in Table 4 and Figure 1.

### 3.5. Evaluation of Body Mass Analysis

Regarding body mass analysis, only metabolic age was significantly higher in patients with symptoms [53.81 (±15.24) vs. 45.68 (±15.48), *p* = 0.02] in comparison to controls. Statistically significant differences between Fat (%), Fat (kg), FFM, TBW (kg), TBW (%), ECW, ICW, ECW/TBW × 100%, and BMR were not detected [*p* > 0.05, for all]. The results are listed in Table 5.

### 3.6. Multivariate Analysis

**Figure 2:**
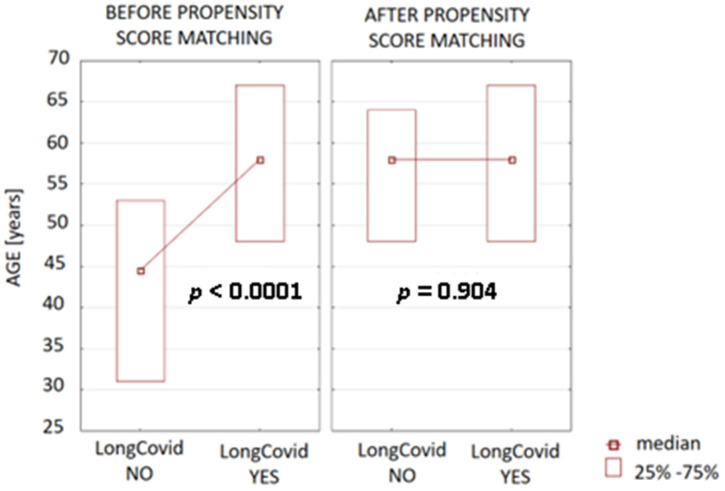
The differences in age between the long COVID and non-long COVID populations before and after matching.

To reduce the bias with age between the long COVID group and the non-long COVID population, one-to-one nearest-neighbor propensity score matching was used. The propensity score estimation was conducted using logistic regression. Next, the patients were matched according to age to verify whether the observed differences between long COVID and non-long COVID groups before matching for the following age-dependent parameters, i.e., A, HR_max_, VO_2max_, FEV1, FVC, FEV1/FVC [%], as well as RER and GFR, will present similar associations after age correction. The difference in age in long COVID and non-long COVID groups before and after matching is presented in Figure 2.

The analysis indicated that the long COVID and non-long COVID age-matched populations did not differ in the following parameters: HR_max_, VO_2max,_ and RER. However, the remaining analyzed parameters differ significantly between the analyzed groups (Table 6).

Parameters with a *p*-value < 0.05 in the univariate analysis were entered into the multivariate analysis using the logistic regression analysis. This analysis pointed out that FEV1/FVC [%] higher than 102 is associated with a high chance of the occurrence of long-term symptoms after SARS-CoV-2 infection (OR = 6.27, 95% Cl: 2.64–14.86; *p* < 0.001). Results are presented in Table 7.

## 4. Discussion

As far as we can tell, the presented study is one of the first analyses of the relationship between echocardiographic, spiroergometric parameters, and hydration status in patients with long COVID symptoms. Patients with long COVID symptoms presented with significantly higher age, metabolic age, LA diameter, LVMI, A velocity, E/E’, and lower E/A compared to the control group. Several teams of researchers have investigated the long-term effects of COVID-19 [11].

Tudoran et al. enrolled 150 patients with no cardiovascular disease, treated them as COVID-19 patients for 4 to 12 weeks before the inclusion of this study and assessed the cardiovascular condition using TTE. The 38 patients (approximately 25%) have found signs of heart disease, including pulmonary hypertension (9%), decreased left ventricular function (8%), diastolic dysfunction (14%), and/or evidence of pericarditis (10%) [22]. In another study, after the diagnosis of COVID-19, the authors detected RV dilation, increased pulmonary pressure, and biventricular dysfunction [23]. Our findings also revealed that LA diameter and LVMI were higher in the study group than in the control group. LA dilation is part of the heart remodeling process in various cardiovascular diseases and is associated with a worse outcome [24]. This abnormality may lead to blood stasis and the formation of thrombi. Furthermore, LA enlargement can indicate other risk factors for strokes and deaths, such as atrial fibrillation, structural heart disease, hypertension, or increased left ventricular mass [11,25]. Our next results show that long COVID patients presented with lower FVC, FEV1, VO_2max_, RER, and higher FEV1/FVC% in comparison to healthy controls. There are a number of studies investigating changes in pulmonary function in patients post-COVID-19. Fumagalli et al. assessed respiratory function at the time of clinical recovery, 6 weeks, 6 months, and 12 months after discharge in patients surviving COVID-19 pneumonia. They revealed that COVID-19 pneumonia may result in significant alterations in lung function, with a mainly restrictive pattern, partly persisting at 6 weeks after recovery from the acute phase but significantly improving during a 12-month follow-up period [26]. Some authors used CPET for the evaluation of long COVID symptoms. In the study by Mancini et al., 58.5% of patients 3 months after SARS-CoV-2 infection had decreased peak VO_2_ in CPET [27]. Durstenfeld et al. conducted a meta-analysis to estimate the differences in exercise capacity among individuals with and without long COVID symptoms. Based on a meta-analysis of nine studies, including 464 symptomatic and 359 asymptomatic patients, the mean peak VO_2_ was −4.9 (95% CI, −6.4 to −3.4) mL/kg/min [28]. The laboratory results in our study showed that patients with long COVID symptoms had lower rates of RBC and GFR and higher levels of glucose and hsTnT. The mentioned parameters are statistically significant, but in clinical practice, they may not be important. However, there are various studies concerning biochemical and hematological abnormalities in patients post-COVID-19. Kubankova et al. found significant phenotypic changes in the RBCs of recovered COVID-19 patients. RBCs are less deformable, smaller, and more heterogeneous in size and deformation [29]. Another study revealed that patients who survived COVID-19 were at greater risk of kidney dysfunction in the post-acute phase of the disease [30]. Some studies also demonstrated the risk of developing hyperglycemia and diabetes after SARS-CoV-2 infection [8,31]. Troponin T is a part of the troponin complex, which is composed of proteins integral to the contraction of skeletal and heart muscles. Cardiac troponin T (cTnT) is the preferred biochemical marker for myocardial cell necrosis [32]. Measurement of the hs-cTnT may provide strong prognostic information in patients with acute coronary syndromes, stable coronary artery disease, heart failure, and even in the general population. Several studies detected higher levels of hsTnT in COVID-19 patients. In one meta-analysis, the authors showed that elevated troponin was associated with mortality rates in patients with COVID-19, with 55% sensitivity and 80% specificity [33]. The results of several logistic regression models independently associated with the long-term symptoms of COVID were FEV1/FVC%. In a study conducted by Daitch et al., 2333 participants who recovered from COVID-19 were evaluated over an average of five months [146 days (95% CI 142–150)] after the initial COVID-19. The average age was 51 years, and 20% were over 65 years. Older adults are more likely to develop symptoms, and the most common symptoms are fatigue (38%), followed by diarrhea (30%). They complained of coughing and arthritis and were more likely to undergo abnormal chest imaging and lung function tests [34].

Long COVID can significantly impact a person’s quality of life, and its management often requires a multidisciplinary approach involving healthcare professionals from various specialties. Research is ongoing to better understand the condition, develop effective treatments, and support individuals affected by long COVID. In one study, the authors examined the heterogeneity of adoption and use of U09.9, the ICD-10-CM code for “Post COVID-19 condition, unspecified.” The research identifies common co-occurring diagnoses, categorizing them into cardiopulmonary, neurological, gastrointestinal, and comorbid conditions. It also reveals a demographic skew, with a higher representation of female, White, non-Hispanic individuals residing in areas with low poverty and unemployment rates among patients diagnosed with long COVID [35]. Izzo et al. highlighted the role of microRNA in cardiovascular complications associated with COVID-19, examining their potential utility as biomarkers, prognostic indicators, and targets for therapeutic interventions [36].

According to the results of the studies mentioned above, it is important to emphasize the role of vaccination against SARS-CoV-2. Vaccines help prevent severe illness, hospitalization, and death caused by COVID-19 [37]. They also contribute to reducing the transmission of the virus within communities. By achieving widespread vaccination coverage, we can establish herd immunity, protecting vulnerable populations and allowing societies to safely return to normalcy [38].

The presented study has potential limitations, including a small study population (146 participants). This study design was limited regarding the evaluation of the effects of used medications. In addition, only patients who were able to perform CPET were enrolled. We were also unable to measure total lung capacity (TLC) and diffusion lung capacity for carbon monoxide (DLCO). Furthermore, we assessed TTE only at rest. Some echocardiographic parameters, such as left atrial strain, were not obtained. These data need to be interpreted with caution. Therefore, future studies, including the measurement of TLC and DLCO in a larger post-COVID population, are recommended.

The strengths of our study are that this is one of the first studies to evaluate the utility of selected echocardiographic, laboratory, and spiroergometric parameters in differentially diagnosing patients presenting the symptoms of long COVID.

## 5. Conclusions

In conclusion, an FEV1/FVC% higher than 102 is associated with a high chance of the occurrence of long-term symptoms after SARS-CoV-2 infection. Persistent symptoms of long COVID can mimic those of cardiovascular disease.

## Figures and Tables

**Table 1:** Evaluation of basic characteristics among the investigated groups.

Parameter	Patients with Symptoms n = 44	Patients without Symptoms n = 102	*p*
Basic characteristics			
Male sex	41%	36%	0.26
Age	(48.00–67.00), 58.00 *	(31.00–53.00), 44.50 *	<0.0001
BMI (kg/m^2^)	(23.90–31.12), 27.11 *	(23.85–31.16), 26.50 *	0.62
BSA (m^2^)	(1.73–2.01), 1.86 *	(1.78–2.11), 1.91 *	0.35
SBP (mmHg)	(124.00–140.00), 130.00 *	(125.50–147.00), 134.50 *	0.15
DBP (mmHg)	(76.00–88.00), 80.00 *	(74.00–90.00), 85.50 *	0.26
HR	(70.00–80.00), 72.00 *	(68.00–83.50), 73.50 *	0.90
HA	54%	46%	0.80
Dyslipidemia	52%	48%	0.40
DM 2	31%	69%	0.21
Nicotinism	37%	63%	0.78
Obesity	55%	45%	0.75

*—median; values with a non-normal distribution are expressed as median (range) values. Values with normal distributions are expressed as the mean ± standard deviation (SD). BMI—body mass index; BSA—body surface area; SBP—systolic blood pressure; DBP—diastolic blood pressure; HR—heart rate; HA—arterial hypertension; DM 2—type 2 diabetes mellitus.

**Table 2:** Evaluation of laboratory tests among the investigated groups.

Parameter	Patients with Symptoms n = 44	Patients without Symptoms n = 102	*p*
Laboratory tests			
hs-cTnT (pg/mL)	(3.20–9.00), 6.10 *	(3.00–6.20), 3.90 *	0.04
NT-proBNP (pg/mL)	(42.00–125.00), 86.00 *	(29.00–123.00), 61.00 *	0.051
RBC (10^6^/uL)	(4.13–4.80), 4.42 *	(4.32–5.08), 4.62 *	0.01
Hemoglobin (g/dL)	(12.60–14.50), 13.30 *	(13.00–15.30), 13.90 *	0.07
PLT (10^3^/uL)	218.87 (±54.59)	223.11 (±54.56)	0.67
Creatinine (mg/dL)	(0.67–0.89), 0.79 *	(0.66–0.90), 0.77 *	0.87
GFR (mL/min/1.73 m^2^)	(76.10–98.80), 88.25 *	(81.80–111.30), 94.90 *	0.03
Urea (mg/dL)	35.24 (±11.26)	31.75 (±9.36)	0.07
Glucose (mg/dL)	(86.00–99.00), 91.50 *	(84.50–93.00), 90.00 *	0.03
ALT (U/L)	(18.00–29.00), 22.50 *	(14.50–34.00), 23.00 *	0.93
ASP (U/L)	(24.00–30.00), 26.50 *	(23.50–38.00), 27 *	0.37
CRP (mg/dL)	(0.50–0.50), 0.50 *	(0.50–0.50), 0.50 *	0.82
D-dimer (ng/mL)	(186.00–450.00), 279.00 *	(197.00–439.00), 276.00 *	0.82
TC (mg/dL)	171.32 (±40.71)	173.55 (±45.88)	0.77
LDL (mg/dL)	94.54 (±34.86)	96.80 (±29.01)	0.71
HDL (mg/dL)	(40.00–58.00), 49.50 *	(36.00–59.50), 50.50 *	0.99
TG (mg/dL)	(86.00–163.00), 111.50 *	(80.00–150.00), 101.00 *	0.57
Na (mmol/L)	(138.00–140.00), 139.00 *	(137.50–140.00), 139.00 *	0.20
K (mmol/L)	(4.20–4.60), 4.30 *	(4.10–4.55), 4.30 *	0.74

*—median; values with a non-normal distribution are expressed as median (range) values. Values with normal distributions are expressed as the mean ± standard deviation (SD). hs-cTnT—high-sensitivity cardiac troponin; NT-proBNP—N-terminal prohormone of brain natriuretic peptide; RBC—red blood cells; PLT—thrombocytes; GFR—glomerular filtration rate; ALT—alanine aminotransferase; AST—aspartate aminotransferase; CRP—c-reactive protein; TC—total cholesterol; LDL—low-density lipoprotein; HDL—high-density lipoprotein; TG—triglycerides; Na—serum natrium; K—serum potassium.

**Table 3:** Evaluation of selected echocardiographic parameters among the investigated groups.

Parameter	Patients with Symptoms n = 44	Patients without Symptoms n = 102	*p*
Echocardiography			
LVID d (mm)	(45.00–53.00), 48.00 *	(43.00–53.00), 46.00 *	0.16
IVS d (mm)	(9.00–11.00), 10.00 *	(9.00–11.00), 9.00 *	0.76
LVPW d (mm)	(8.00–10.00), 9.00 *	(8.00–10.00), 9.00 *	0.75
LA (mm)	(34.00–42.00), 37.00 *	(32.00–39.00), 35.00 *	0.04
LAV (mL)	(47.00–81.00), 63.00 *	(41.50–73.00), 53.50 *	0.23
LAVi (mL/m^2^)	(26.55–40.98), 33.30 *	(23.00–38.10), 28.65 *	0.10
RA (cm^2^)	(13.50–18.50), 16.10 *	(13.00–20.00), 16.15 *	0.86
RVOT d (mm)	31.45 (±4.38)	30.57 (±4.87)	0.30
AB (mm)	(30.00–36.00), 32 *	(30.00–37.00), 33.00 *	0.97
AA (mm)	(29.00–36.00), 32.00 *	(28.00–36.00), 31.00 *	0.56
MPA (mm)	(18.00–21.00), 19.50 *	(18.00–21.00), 19.00 *	0.78
IVC (mm)	(4.50–9.00), 6.50 *	(4.00–10.00), 6.00 *	0.79
LVMI (g/m^2^)	(71.00–98.00), 83.00 *	(61.00–98.00), 74.00 *	0.04
LVEF (%)	(56.00–65.00), 62.00 *	(59.00–67.00), 62.00 *	0.37
EDV (cm^3^)	(71.00–103.00), 89.00 *	(75.00–105.00), 89.50 *	0.88
ESV (cm^3^)	(25.00–42.00), 34.00 *	(25.00–44.00), 33.00 *	0.79
TAPSE (mm)	23.31 (±3.83)	22.83 (±3.98)	0.50
TDE S’ (cm/s)	(12.00–16.00), 14.00 *	(12.00–15.00), 13 *	0.10
GLPS (%)	19.68 (±1.91)	19.90 (±1.96)	0.62
E (cm/s)	(63.00–85.00), 72.00 *	(60.00–90.00), 79.00 *	0.49
A (cm/s)	(60.00–83.00), 69.00 *	(51.00–74.00), 64.00 *	0.01
E/A	(0.80–1.22), 1.05 *	(0.95–1.67), 1.31 *	0.01
E/E’	(6.00–8.95), 7.35 *	(5.50–7.10), 6.05 *	0.01
Dec (ms)	(167.00–245.00), 205.00 *	(175.00–239.00), 194.00 *	0.79
Ats (ms)	(114.00–145.00), 133.00 *	(118.00–145.00), 133 *	0.68

*—median; values with a non-normal distribution are expressed as median (range) values. Values with normal distributions are expressed as the mean ± standard deviation (SD). LVID d—left ventricular internal diameter end diastole; IVS d—interventricular septum end diastole; LVPW d—left ventricular posterior wall end diastole; LA—left atrial diameter; LAV—left atrial volume; LAVi—left atrial volume index; RA—right atrial area; RVOT—distal right ventricular outflow tract; AB—aortic bulb; AA—ascending aorta; MPA—main pulmonary artery; IVC—inferior vena cava; LVMI—left ventricular mass index; LVEF—left ventricular ejection fraction; EDV—end-diastolic volume; ESV—end-systolic volume; TAPSE—tricuspid annular plane systolic excursion; TDE S’—tissue Doppler echocardiography; GLPS—global peak systolic strain; E—early diastolic filling velocity; A—late diastolic filling velocity; E/A—ratio of early to late diastolic transmitral flow velocity; E/E’—ratio of peak velocity of early diastolic transmitral flow to peak velocity of early diastolic mitral annular motion as determined by pulsed wave Doppler; Dec—deceleration time; Ats—acceleration time.

**Table 4:** Evaluation of spiroergometry among the investigated groups.

Parameter	Patients with Symptoms n = 44	Patients without Symptoms n = 102	*p*
Spiroergometry			
Exercise time (s)	(384.00–662.00), 512.50 *	(412.00–719.00), 559.00 *	0.38
Level of effort (Wat)	(100.00–150.00), 125.00 *	(100.00–171.00), 125.00 *	0.59
HR max	146.63 (±20.46)	132.87 (±33.61)	0.006
Peripheral SBP max (mmHg)	(140.00–190.00), 160.00 *	(140.00–195.00), 160.00 *	0.38
Peripheral DBP max (mmHg)	(70.00–90.00), 80.00 *	(75.00–90.00), 80 *	0.86
FEV1 (L)	(2.55–3.54), 2.90 *	(2.79–3.71), 3.25 *	0.04
FVC (L)	3.65 (±0.89)	4.32 (±1.02)	<0.0001
FVC (%)	106.45 (±17.83)	107.00 (±15.27)	0.88
FEV1/FVC	(77.00–88.00), 83.00 *	(74.00–85.00), 80.00 *	0.051
FEV1/FVC (%)	(99.00–112.00), 106.50 *	(90.00–105.00), 100.00 *	0.0002
FEF 25–75 (L/s)	2.79 (±1.27)	3.17 (±0.98)	0.14
RER	(1.01–1.11), 1.08 *	(1.05–1.12), 1.10 *	0.04
VO_2max_ (mL/min/kg)	(16.00–25.00), 21.00 *	(19.00–29.00), 23.00 *	0.04
VO_2_AT (mL/min/kg)	(11.00–17.00), 14.00 *	(13.00–20.00), 15.50 *	0.07
Peak VO_2max_ (L)	(1.29–1.90), 1.58 *	(1.38–2.23), 1.77 *	0.07
VE/VCO_2_ slope	(26.10–33.90), 29.70 *	(25.40–32.00), 28.05 *	0.26

*—median; values with a non-normal distribution are expressed as median (range) values. Values with normal distributions are expressed as the mean ± standard deviation (SD). FEV1—forced expiratory volume in one second; FVC—forced vital capacity; FEV1/FVC—ratio of forced expiratory volume in one second to forced vital capacity; FEF 25–75%—forced expiratory flow over the middle one half of the FVC; RER—respiratory exchange ratio; VO_2max_—the maximum amount of oxygen the body can utilize during a specified period of usually intense exercise; VO_2_AT—oxygen uptake at anaerobic threshold per kilogram; peak VO_2_—highest respiratory oxygen uptake (VO_2_) achieved by the subject during the maximal exercise; VE/VCO_2_ slope—the minute ventilation/carbon dioxide production slope.

**Table 5:** Evaluation of body mass analysis among the investigated groups.

Parameter	Patients with Symptoms n = 44	Patients without Symptoms n = 102	*p*
Body mass analysis			
Fat (%)	30.00 (±6.40)	30.21 (±7.69)	0.89
Fat (kg)	(17.40–29.10), 23.50 *	(19.50–33.95), 24.70 *	0.44
FFM (kg)	(47.40–60.40), 53.60 *	(47.50–65.50), 56.65 *	0.29
TBW (kg)	(33.85–44.65), 39.15 *	(34.30–46.40), 41.00 *	0.49
TBW (%)	51.00 (±5.10)	50.06 (±6.81)	0.46
ECW (kg)	(15.15–19.45), 17.20 *	(15.40–19.55), 18.15 *	0.52
ICW (kg)	(19.05–25.50), 22.00 *	(19.30–27.30), 22.45 *	0.44
ECW/TBW × 100%	43.58 (±2.67)	43.25 (±3.65)	0.62
Metabolic age	53.81 (±15.24)	45.68 (±15.48)	0.02
BMR (kcal)	(1279.00–1701.00), 1493.00 *	(1395.50–1888.50), 1561.50 *	0.08

*—median; values with a non-normal distribution are expressed as median (range) values. Values with normal distributions are expressed as the mean ± standard deviation (SD). FFM—fat-free body mass; TBW—total body water; ECW—extracellular water; ICW—intracellular water; ECW/TBW%—ratio of extracellular water to total body water; BMR—basal metabolic rate.

**Table 6:** Significant differences among the investigated groups after propensity score matching.

Parameter	Patients with Symptoms n = 44	Patients without Symptoms n = 102	*p*
A (cm/s)	(60.00–83.00), 69.00 *	(58–78), 67 *	0.04
FVC (L)	(3.08–4.27), 3.53 *	(3.80–4.30), 4.26 *	0.0001
FEV1 (L)	(2.55–3.54), 2.90 *	(2.91–3.53), 3.11 *	0.045
FEV1/FVC (%)	(99.00–112.00), 106.50 *	(95.00–102.00), 96.00 *	<0.0001
GFR (mL/min/1.73 m^2^)	(76.10–98.80), 88.25 *	(79.88–109.80), 93.20 *	0.04

*—median; values with a non-normal distribution are expressed as median (range) values. Values with normal distributions are expressed as the mean ± standard deviation (SD). A—late diastolic filling velocity; FVC—forced vital capacity; FEV1—forced expiratory volume in one second; FEV1/FVC—ratio of forced expiratory volume in one second to forced vital capacity; GFR—glomerular filtration rate.

**Table 7:** Multivariate analysis—stepwise logistic regression.

Variable	OR	95% CI for OR		*p*
		Lower Limit	Upper Limit	
FEV1/FVC% > 102%	6.27	2.64	14.86	<0.001

FEV1/FVC—ratio of forced expiratory volume in one second to forced vital capacity.

## Data Availability

Individual participant data that underlie the results reported in this article after deidentification (text, tables, figures, and appendices) as well as the study protocol will be available for researchers who provide a methodologically sound proposal. Proposals may be submitted after 9 months and up to 36 months following article publication.
